# A mobile health + health coaching application for the management of chronic non-cancer pain in older adults: Results from a pilot randomized controlled study

**DOI:** 10.3389/fpain.2022.921428

**Published:** 2022-07-25

**Authors:** Usha Kaul, Clara Scher, Charles R. Henderson, Patricia Kim, Mette Dyhrberg, Vanessa Rudin, Millie Lytle, Nicole Bundy, M. Carrington Reid

**Affiliations:** ^1^Division of Geriatrics and Palliative Medicine, Weill Cornell Medical Center, New York, NY, United States; ^2^Rutgers School of Social Work, New Brunswick, NJ, United States; ^3^College of Human Ecology, Cornell University, Ithaca, NY, United States; ^4^Mymee Inc., New York, NY, United States

**Keywords:** pain management, digital technology, mobile health, symptom tracking, health coaching, older adults

## Abstract

**Introduction:**

The rapid growth of mobile health (mHealth) devices holds substantial potential for improving care and care outcomes in aging adults with chronic non-cancer pain (CNCP), however, research evaluating these devices in older adults remains limited.

**Objective:**

To ascertain the feasibility and preliminary efficacy of an mHealth intervention (Mymee) that combines symptom, diet, and behavior tracking *via* a smartphone application with data analytics to detect associations between symptoms and lifestyle factors along with weekly health coaching sessions to mitigate CNCP in adults 55 years of age and older.

**Methods:**

Participants (*N* = 31) in this pilot study were recruited from one primary care practice in New York City and randomized to an intervention [app + up to 12 health coaching sessions (scheduled approximately once weekly) + usual care] or a control (app + usual care) arm. Feasibility measures included recruitment (proportion of eligible persons who enrolled) and retention rates (proportion of subjects completing a follow-up assessment) as well as adherence with the weekly coaching sessions and logging daily data on the app. Efficacy outcomes (e.g., pain intensity, self-efficacy, disability, anxiety) were assessed at baseline and follow-up (~16 weeks after baseline). Descriptive statistics were obtained and general linear mixed models used for primary analyses.

**Results:**

Participants had a mean (standard deviation) age of 67.32 (9.17) and were mostly female (61%). Feasibility outcomes were mixed as evidenced by recruitment and retention rates of 74% and 65%, respectively. The mean number of weekly coaching sessions attended by intervention participants was 6.05 (SD = 5.35), while the average number of days logging data on the app was 44.82 (34.02). We found a consistent trend in favor of the intervention, where pain intensity, affect, and quality of life measures improved considerably more among intervention (vs. control) participants. Finally, the proportion of participants with GAD-7 scores at follow up decreased by 0.35 to 0, whereas controls did not change, a significant effect in favor of the intervention (*p* = 0.02).

**Conclusions:**

This study supports the need for future research that seeks to enhance feasibility outcomes and confirm the efficacy of the Mymee intervention among aging adults with CNCP.

## Introduction

The population of Americans ages 65 and older adults will reach 95 million by 2060, nearly doubling in size from 2018 (1). Older adults are disproportionately affected by chronic diseases, many of which have pain as a primary symptom (2). Chronic non-cancer pain (CNCP) is a common, morbid, and costly condition among older adults (3). As the number of older adults with CNCP continues to rise and the need for virtual methods of healthcare delivery increases given the ongoing COVID-19 pandemic, researchers have begun to examine whether mobile health devices (hereafter referred to as mHealth) can enhance the management of pain in patients in this age group (4).

mHealth, as defined by the World Health Organization, includes “medical and public health practice supported by mobile devices, such as mobile phones, patient monitoring devices, personal digital assistants (PDAs), and other wireless devices” (5). Since 2013, the number of older adults who report owning a smartphone has more than doubled, expanding prospects for delivering mHealth applications to aging adults (6). Previous research has shown that older adults are interested in using mHealth technologies to manage pain and other chronic disease-related symptoms (7, 8). Further, mHealth technologies have shown promise as a means of increasing patient-to-provider communication, encouraging pain self-management and medication adherence, as well as motivating positive behavior change (8).

Although the scope of mHealth applications and tools continues to expand, research examining the feasibility and efficacy of these technologies as a means of mitigating CNCP in older adults remains limited. One systematic review examined the effects of mHealth interventions on the management of CNCP in older adults and identified just 10 studies (9). The studies were largely qualitative, and the authors concluded that research investigating mHealth tools to manage CNCP in older adults remains scant (9).

Health coaching constitutes an evidenced-based method for helping individuals adopt health enhancing behaviors (10). Using a patient-oriented approach, health coaches help patients effect behavior change (e.g., alterations in diet and exercise) in order to improve their overall health and quality of life (10). One recent study that examined the effects of health coaching intervention in individuals with CNCP found clinically and statistically significant improvements in pain intensity and pain interference (11).

The current pilot randomized-controlled study sought to examine the feasibility and preliminary efficacy of an mHealth application that combines (1) symptom, diet, and behavior tracking via a smartphone application with data analytics to detect associations between symptoms and lifestyle factors, along with (2) weekly health coaching sessions to mitigate pain in community-dwelling adults ages 55 and above.

The intervention, described in more detail below, was designed by Mymee, Inc. as an adjunctive non-pharmacologic method to help identify and mitigate triggers of disease flares in patients with autoimmune disease. In a prior study, the intervention improved health-related quality of life in patients with systemic lupus erythematosus when delivered along with usual care (12). Given that many patients with CNCP face similar symptoms (e.g., pain, stiffness, depressed mood, acute-on-chronic pain flares) as patients with rheumatologic disease and endorse similar barriers to successful self-management of pain (13–15), this study sought to determine the feasibility and preliminary efficacy of the intervention in a small sample of older adults with CNCP.

## Methods

### Design

In this pilot investigation the study goals were to examine indicators of the intervention's feasibility (recruitment, retention) and adherence with the elements of the intervention (to include logging of data and meeting weekly with the health coach) and preliminary efficacy. No formal power calculations were conducted, consistent with recent guidelines for conducting pilot feasibility studies (16). The goal was to recruit ~15 participants into each study arm.

### Recruitment methods and study setting

Participants were recruited by phone and in person at a Weill Cornell Medicine/New York Presbyterian ambulatory care practice serving over 5,000 older adults. Methods of recruitment included approaching persons waiting for their scheduled appointments in the practice's waiting room, posting study flyers in the practice's waiting area, and reminding practice physicians to refer prospective participants when appropriate. Prior participants from other pain studies who expressed interest in participating in future research were also contacted by phone to determine their interest in participating. The study took place between September 2018 and December 2019.

### Participants

Subjects included individuals who: (1) were ages 55 years or older; (2) endorsed experiencing CNCP on most days during the past 3 months; (3) reported an average pain level of 4 or greater on a 0-to-10 scale; (4) evidenced some degree of pain-related interference, defined as experiencing at least one day in the past month where pain limited their everyday activities; and (5) had access to an iPhone, Android phone, iPad, or Android tablet. Exclusion criteria included any planned surgery during the study period (~12 weeks), plans to travel from their home within the United States for more than 2 weeks during the study period, self-reported severe auditory and visual deficits, and current (or anticipated) participation in another study.

### Study procedures

The Weill Cornell Institutional Review Board approved the study, and all participants provided written informed consent. At the initial visit, a research assistant administered the baseline assessment (described below) and helped participants download the Mymee application on their smartphones/tablets. After completing the baseline assessment, participants were randomly assigned to the intervention or control group using an online random number generator with set parameters, odd numbers being assigned to usual care and even numbers being assigned to intervention.

Participants randomized to active treatment received training in how to use the app to track symptoms (e.g., pain), food intake, behaviors that triggered or reduced pain, and were encouraged to communicate any health issues they felt were important to share with their health coach. These participants received access to the application along with telephone coaching sessions scheduled approximately once weekly (up to a maximum of 12 sessions) with a Mymee health coach. The health coach reviewed weekly tracking data provided by each participant and made personalized recommendations based on these data. The control group received access to the app without any coaching sessions.

Two weeks after program conclusion (week 14), participants completed a follow-up assessment either in person or over the phone. Due to difficulties in getting some of the follow-up assessments scheduled, a small number (*n* = 5) took place in week 15 or 16. Efficacy measures administered at the baseline assessment were re-administered at this time. Each participant received a $25 compensation at both the baseline and post assessment visits.

### Mymee intervention

The Mymee platform combines self-tracking technology, analytics, and tele-coaching. The application allows participants to log symptoms (e.g., pain, stiffness), behavioral and lifestyle factors, food intake, and general notes by providing daily entry selections. Data entry takes approximately 5 minutes a day. Data pertaining to an individual's daily pain levels, dietary intake, exercise and sleep patterns, and water consumption were tracked and stored using the application and through notes taken by Mymee health coaches. There is an established evidence base that supports targeting lifestyle factors such as physical activity, sleep disturbance, and weight reduction (17–20) and a growing evidence base to support targeting nutrition (21–24) in order to reduce pain levels and associated morbidity among individuals living with CNCP.

The data were reviewed weekly by a Mymee health coach who then designed personalized interventions focused on addressing environmental and lifestyle triggers as well as dietary factors, when appropriate, with the goal of providing meaningful improvements in the patient's symptoms (e.g., reduction in pain, improvements in affect) and overall health-related quality of life (12).

Mymee staff receive over 120 h of training in how to engage and work with diverse clients to affect behavior changes.

The health coaching sessions occurred approximately once weekly *via* telephone and lasted up to 30 mins. At the first coaching session, health coaches asked a series of questions regarding the participant's activity level, environmental barriers, bodily functions, food intake, and pain status. At subsequent sessions, Mymee health coaches (1) identified potential pain triggers (based on associations between symptoms and dietary and lifestyle factors as revealed by the tracked data) and recommended lifestyle adjustments for participants to try, (2) inquired whether the recommendations made in previous sessions had any discernible effects, and (3) helped to problem solve if participants had difficulty making the recommended behavioral changes. During the weekly coaching sessions, Mymee coaches worked with intervention participants to effect lifestyle modifications aimed at healthy habit creation and sustainability, sleep quality and timing, optimizing physical activity levels, and building self-efficacy. They also focused on optimizing nutrient intake and food-trigger avoidance.

The coaching model employed by Mymee coaches is based on social cognitive theory and leverages motivational interviewing principles. Mymee coaches work with patients to help set realistic, actionable goals and reinforce behavior change by offering encouragement when goals are met, thereby enhancing patients' self-efficacy. Coaches are trained to explore ambivalence patients often feel in making a behavior change (supporting discrepancies), inquire about motivations for change, express empathy regularly, and use problem-solving skills to help patients overcome barriers to achieving behavior change. Finally, the daily tracking patients perform *via* the app enhances their self-monitoring skills.

### Data collection

#### Feasibility measures

Feasibility measures included: (1) the proportion of eligible persons who enrolled in the study (recruitment rate), (2) the proportion of participants who completed both the baseline and follow-up assessments (retention rate), (3) the number of coaching sessions attended by intervention participants, and (4) the number of days participants logged data on the Mymee app. Intervention arm participants were asked to enter data on a daily basis over the study period, while control participants were instructed to use the app as they saw fit.

#### Efficacy measures

The following measures were administered at baseline and follow-up by trained research assistants. Pain intensity was assessed using a single-item measure (current pain level) that ranged from 0 to 10 (where 10 is worst pain imaginable). Participants' level of pain-related disability was assessed with the Roland-Morris Disability Questionnaire (RMDQ) (25), where higher scores indicate higher levels of pain-related disability (18). The RMDQ was originally used to quantify the degree of disability due to back pain but is increasingly being used to determine pain-related disability in general pain populations (26–29). The Pain Self-Efficacy Questionnaire (PSEQ) (30) was used to evaluate participants' perceived self-efficacy to cope with the consequences of CNCP.

Additional outcomes included the General Anxiety Disorder scale (GAD-7) (31). A variable for the presence of clinically significant anxiety symptoms was created using a GAD-7 cutoff score of 10 or greater (31). To assess levels of both positive and negative emotions, the Positive and Negative Affect Scale (PANAS) was administered (32). Finally, participants completed the Quality-of-Life Enjoyment and Satisfaction Questionnaire (Q-LES-Q-SF) (33) to assess their degree of life satisfaction and enjoyment over the past week.

#### Independent variables

Data were collected on participants' sociodemographic characteristics, including gender, age, race/ethnicity, marital status, years of education, and living arrangement. Finally, the Lawton Activities of Daily Living Scale (ADL) (34) was used to measure participants' ability to perform both basic and instrumental activities of daily living, with higher scores reflecting better overall functioning.

### Qualitative data collection methods

Qualitative data were gathered at the time of the follow-up assessment and during phone interviews with participants who did not complete the second assessment. Open-ended questions were employed to explore intervention participants' likes and dislikes associated with the weekly health coaching sessions and the daily tracking request. Intervention participants were also given an opportunity to share any other issues that they felt would be important for the research team to learn about their participation in the study. Exit interviews were not conducted with control participants. Finally, participants who dropped out of both the intervention and control arms were asked to report on their reason(s) for doing so.

### Qualitative analysis

The transcribed interviews were analyzed using content analysis (35). Two investigators independently reviewed the qualitative data and systematically organized data into a structured format. Codes, categories, and themes were constructed individually and continually revised and reformulated after reviewing each new transcript. No categories or themes were predetermined beforehand. The investigators then met to compare and discuss findings and reconciled any differing themes until there was an agreement on a framework of themes and their definitions.

### Statistical analysis

Descriptive statistics were obtained for the sociodemographic, clinical, and outcome variables at baseline. The core model for evaluating the intervention's potential efficacy for each outcome included treatment (2 levels—control and intervention) and time of assessment (2 levels—baseline and at follow-up) as fixed classification factors, the interaction of these 2 factors, and individuals as levels of a random classification factor. Models that included an *a priori* set of additional independent variables chosen based on the literature and our prior research were also examined and include gender, race/ethnicity (White, Black, or Other), marital status (Married/Partnered, Widowed, Divorced/Separated, Never Married), living arrangement (Alone, With Spouse or Partner, With Others), and education (Some College or Less, College Graduate, Post Graduate Degree) as fixed classification factors and age as a covariate (i.e., quantitative variable).

The interaction of each of the additional variables with treatment and time was also examined—a 3-way interaction of fixed factors for the categorical variables and homogeneity of regressions of the covariate by levels of treatment and time for the covariates (36). There was no coherent significance for interactions, and the final models presented do not include interactions.

Analysis was by general linear mixed models assuming normality with unstructured error. Degrees of freedom were computed using the first-order Kenward-Rogers method (37). The GAD-7 variable was also examined in dichotomous form, with cut-off value of 10 below. Initially, a logistic-linear mixed model with binomial error was considered but the model was numerically ill-behaved, even with various initial solutions and estimation methods, and ultimately we reverted to an assumption of normality.

The key test of the effectiveness of the intervention is the treatment × time interaction. **Table 2** shows least squares means plus standard errors and differences of means and p-values for tests of those differences.

Missing data at follow up were handled by the maximum likelihood estimation of the mixed models. As a sensitivity analysis, models in which cases were restricted to individuals who had complete data at follow up were examined. Results did not differ meaningfully from results of the main analyses, and they are not presented here.

For the intervention sample, the number of sessions attended as a function of each sociodemographic variable, were analyzed in separate models. The primary models the regressions of each outcome on number of sessions were also examined. There was no coherent pattern of better outcomes for greater attendance, and those results are not reported.

## Results

### Sociodemographic characteristics of the study sample

Sample descriptive statistics appear in Table 1. The mean (standard deviation = SD) age of the sample was 67.32 (9.17). Most participants (61%) were female; 45% were non-Hispanic white, 42% were Black (100% self-reported as non-Hispanic Black), and 13% identified as “other.” The mean functional status score in the total sample was 26.00 (SD = 2.63) indicating excellent overall functional status.

**Table 1:** Sociodemographic characteristics of the study sample.

	**Control (*****N*** = **13)**	**Intervention (*****N*** = **18)**	**Total sample (*****N*** = **31)**
	**Mean (SD)/Frequency**	**Mean (SD)/Frequency**	**Mean (SD)/Frequency**
Age	66.86 (9.09)	67.70 (9.49)	67.32 (9.17)
Female	54%	67%	61%
**Race and/or Ethnicity**			
White	54%	39%	45%
Black	38%	44%	42%
Other	8%	17%	13%
**Education**			
Some college or less	31%	50%	42%
College graduate	8%	17%	13%
Post graduate degree	62%	33%	45%
**Marital status**			
Married/partnered	23%	22%	23%
Widowed	23%	17%	19%
Divorced or Separated	15%	44%	32%
Never married	38%	17%	26%
**Living Situation**			
Alone	54%	72%	65%
With Spouse/Partner	23%	17%	19%
With others	23%	11%	16%
**Functional status**			
ADL (0 - 14)	13.07 (1.71)	13.28 (0.96)	13.19 (1.30)
IADL (0 - 14)	13.00 (1.47)	12.67 (1.61)	12.81 (1.54)
Total (0 - 28)	26.08 (3.12)	25.94 (2.31)	26.00 (2.63)

### Feasibility outcomes

**Figure 1:**
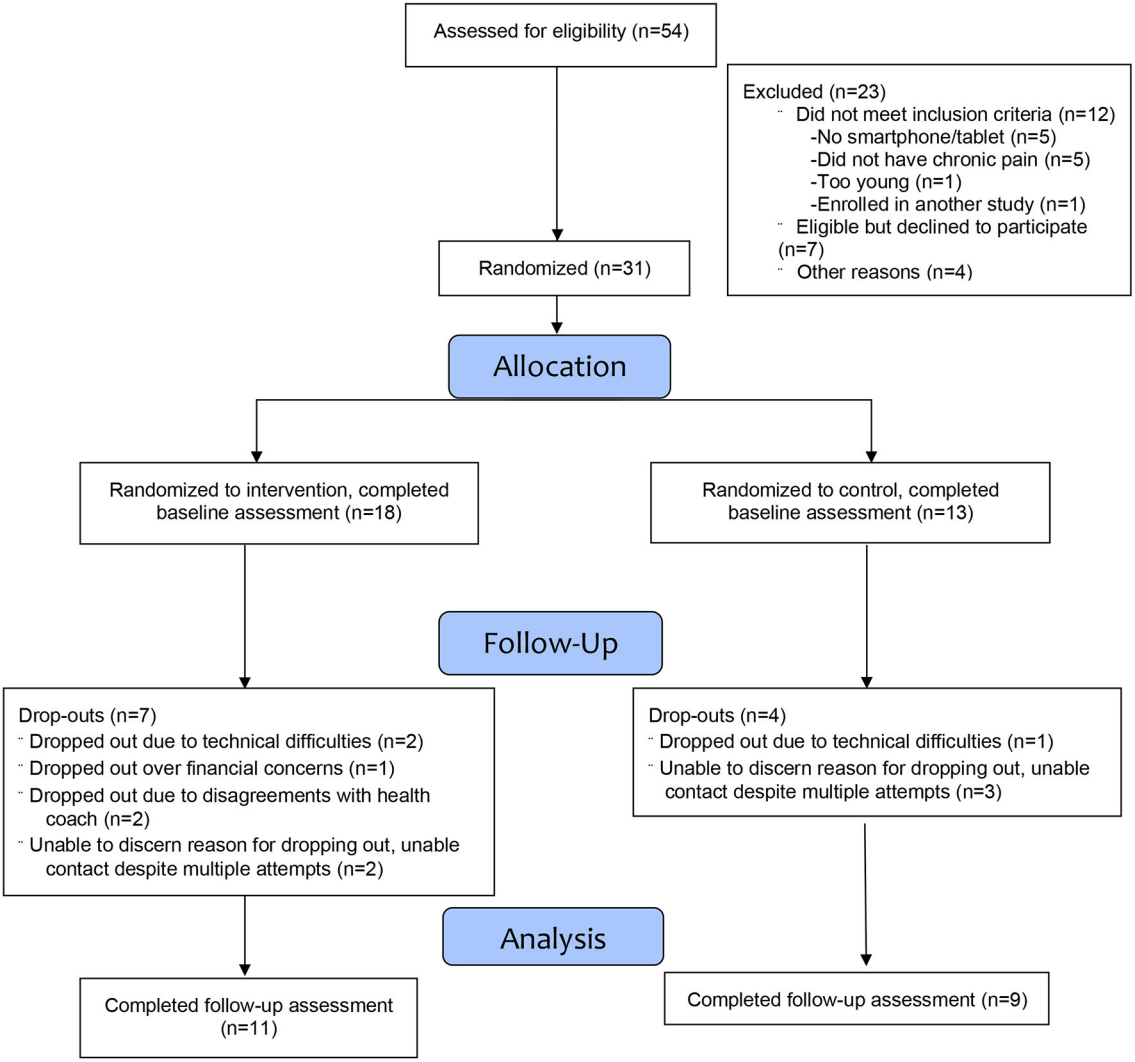
Enrollment and randomization flow diagram.

Of the 54 individuals assessed for eligibility, 42 met the study eligibility criteria and of these, 31 enrolled (see Figure 1), yielding an overall recruitment rate of 74%. Of the 31 individuals who completed the baseline assessment, 20 also completed the follow-up assessment for an overall retention rate of 65%. Of the 11 participants who dropped out, the main reason for doing so was because of little interest in continuing in the study, while a few participants stated that the reason was because they disagreed with the recommended interventions made by the health coach or because of financial concerns (e.g., cost of a dietary supplement that was recommended by a health coach).

Adherence with the weekly coaching sessions was as follows: 7 (39%) attended all 12 sessions, 1 attended 8 sessions and was deemed to have completed all of her goals and therefore did not need to attend the additional 4 sessions, 1 completed 7 sessions, while the remaining 9 (50%) completed 0–3 sessions each. The mean number of weekly coaching sessions attended by intervention participants was 6.05 (SD = 5.35). Of the 7 intervention participants who dropped out, 5 did not attend any of the coaching sessions, 1 drop-out attended 3 sessions and the final drop-out attended 1 session.

The number of sessions attended differed significantly by marital status, such that those who were married or partnered attended an average of 7.5 (4.5) sessions, those who were widowed attended an average of 0 sessions, those who were divorced or separated attended an average of 4 sessions (3.6), and those who were never married attended an average of 10.5 (0.71) sessions.

Among intervention participants, logging data on the Mymee app varied widely, ranging from 4 to 84 days, with a mean of 44.82 (34.02) days. Three intervention participants voiced a preference for logging data using a paper and pencil method that was allowed by the Mymee health coaches. Among control participants, the mean number of days this group logged data on the app was 34.23 (36.72) with a range of 1 to 84 days.

### Efficacy outcomes

The results of the general linear mixed models appear in Table 2. A consistent trend was observed whereby intervention participants achieved superior outcomes relative to control participants. For example, pain intensity scores decreased by 31% in intervention participants but only by 9% among control arm participants. Pain self-efficacy scores also increased by 29% in the intervention group vs. 16% in the control group. Further, pain-related disability scores decreased by 22% among intervention participants (vs. by 9%) in the control arm. Anxiety symptoms decreased by 55% in the intervention arm vs. 22% among control arm participants (Table 2). Finally, the proportion of participants with GAD-7 scores at follow up decreased by 0.35 to 0, whereas controls did not change, a significant effect in favor of the intervention (*p* = 0.02).

**Table 2:** Examination of the effects of the intervention.

	**Baseline** **estimate (SE)**	**Follow-up** **estimate (SE)**	**Follow-up —baseline** **(*****p*****-value)**
Pain intensity (0–10)			
Control	4.90 (1.08)	4.53 (1.10)	−0.35 (0.605)
Intervention	3.83 (0.92)	2.62 (0.10)	−1.21 (0.136)
Intervention—control	−1.07 (1.18)	−1.93 (1.26)	*p* = 0.412
Pain related disability 24-item (0–24)
Control	13.70 (3.33)	12.42 (3.35)	−1.28 (0.249)
Intervention	11.84 (2.85)	9.02 (2.90)	−2.81 (0.042)
Intervention—control	−1.86 (3.58)	−3.40 (3.64)	*p* = 0.367
Pain self-efficacy (0–60)			
Control	31.61 (5.68)	36.67 (5.62)	5.06 (0.284)
Intervention	37.61 (4.66)	48.59 (5.28)	10.98 (0.047)
Intervention—control	6.01 (6.23)	11.92 (6.65)	*p* = 0.400
General anxiety disorder-7 total (0–21)			
Control	7.07 (2.71)	5.53 (2.75)	−1.54 (0.350)
Intervention	6.87 (2.31)	3.07 (2.49)	−3.80 (0.057)
Intervention – Control	−0.20 (2.96)	−2.46 (3.13)	*p* = 0.372
General Anxiety Disorder-7 310			
Control	0.16 (0.19)	0.18 (0.19)	0.02 (0.857)
Intervention	0.32 (0.16)	−0.03 (0.17)	−0.35 (0.008)
Intervention—Control	0.16 (0.21)	−0.20 (0.22)	*p* = 0.028
Positive and negative affect scale: positive (10–50)
Control	32.32 (3.05)	32.35 (3.25)	0.03 (0.990)
Intervention	35.10 (2.61)	38.06 (2.91)	2.96 (0.276)
Intervention—Control	2.79 (3.36)	5.71 (3.68)	*p* = 0.421
Positive and negative affect scale negative (10–50)
Control	21.28 (3.60)	16.93 (3.81)	−4.35 (0.112)
Intervention	19.21 (3.07)	17.71 (3.39)	−1.50 (0.607)
Intervention—control	−2.07 (3.94)	0.78 (4.28)	*p* = 0.475
Quality of life (0–80)			
Control	55.97 (5.00)	56.94 (5.26)	0.97 (0.745)
Intervention	62.81 (6.13)	68.58 (6.31)	5.77 (0.129)
Intervention—control	6.84 (6.50)	11.64 (6.85)	*p* = 0.318

Results are based on a model that included treatment (2 levels—control and intervention) and time of assessment (2 levels—baseline and, follow-up) as fixed classification factors, the interaction of these 2 factors; gender, race/ethnicity (White, Black, or Other), marital status (Married/Partnered, Widowed, Divorced/Separated, Never Married), living arrangement (Alone, With Spouse or Partner, With Others), and education (Some College or Less, College Graduate, Post Graduate Degree) as additional fixed classification factors; age as a covariate; and individuals as levels of a random classification factor.

A higher score on the measures used to assess pain self-efficacy, quality of life and positive affect indicates a better outcome; while a higher score on the measures used to assess pain intensity, pain-related disability, anxiety level and negative emotions indicates a worse outcome.

### Qualitative outcomes

Intervention participants shared positive experiences about their participation in the study. Analysis of the exit interview data revealed 3 major themes documenting positive aspects of the experience: (1) participants valued the support/encouragement received by the health coaches, (2) participants' self-monitoring behaviors were enhanced, and (3) the app was easy to use. Intervention participants described their interactions with the health coaches using term like “inspired me,” “I really liked the encouragement [name of health coach] provided on a weekly basis,” “I really liked her upbeatness and level of attentiveness” (theme 1), while others reported that the tracking and weekly health coaching sessions helped them to understand connections between lifestyle factors and their pain (theme 2). As one participant noted “staying away from tomato sauce really helped my pain.” Finally, most intervention participants noted that the app was “user friendly” while another said “it was very easy to use” (theme 3).

Four themes that reflected participants' dissatisfaction with various elements of the interventions were identified. These themes emerged during the exit interviews with intervention participants and in phone interviews with both intervention and control participants that dropped out of the study. Dissatisfaction themes included (1) skepticism that lifestyle modifications could mitigate pain, (2) desire for more input from intervention participants' primary care providers about the targets selected for intervention, (3) financial barriers, and (4) technical literacy/time constraint issues. Several intervention participants stated that they found it hard to believe that changing their lifestyle would lead to decreased pain levels (theme 1). As one intervention participant reported “I didn't see how changing my diet was going to have an impact on my neuropathic pain,” while another participant stated, “I didn't feel comfortable (making the lifestyle change) without running it by my doctor first” (theme 2). Several intervention participants were asked to begin using nutritional supplements that they found hard to continue because of the costs (theme 3). Finally, several participants (both intervention and control) reported that they did not find the app easy to use or did not like that they had to report data on a daily basis (theme 4).

## Discussion

This study sought to examine the feasibility and preliminary efficacy of an mHealth intervention that combines symptom, diet, and behavior tracking through a smartphone application coupled with data analytics to detect associations between symptoms and lifestyle factors along with weekly health coaching sessions to mitigate CNCP among community-dwelling aging adults. Our investigation adds to the literature by demonstrating issues with the feasibility of the multicomponent intervention evaluated in the current study, but also highlights its potential value when managing CNCP among aging adults.

Our methods led to an acceptable recruitment rate with approximately three-quarters of eligible individuals enrolling in the study, which supports prior research showing that older adults with pain are interested in digital applications (8, 9, 38). Despite this success, the ability to retain participants was much lower than anticipated as evidenced by a dropout rate of 35%. Adherence with the weekly health coaching sessions was also disappointing. Qualitative data analyses identified factors that likely contributed to these outcomes. Several intervention participants reported skepticism that lifestyle modification could lead to reduced pain. Future studies could examine whether providing more education about the evidence demonstrating relationships between lifestyle modification and pain mitigation could potentially enhance adherence and retention outcomes. Another theme identified during the exit interviews was that several intervention participants voiced a desire to get input from their primary care provider before making a lifestyle change. This barrier also appears modifiable as future research could examine various ways in which health coaches delivering the intervention could seek input from (or partner with) participants' primary care providers. Such initiatives could serve to reassure participants about the safety and potential benefit of the recommended lifestyle changes. Several participants dropped out over financial concerns (i.e., cost of recommended nutritional supplements was an economic barrier), while others dropped out over technical literacy issues/time constraints, i.e., did not like having to log data on a daily basis. Helping to cover the costs of dietary supplements recommended by the Mymee health coaches warrants future research as do efforts to provide more training to individuals with limited technical literacy. Increasing the education participants receive (e.g., in the first session) about why tracking data are critical to the success of the program and ensuring that participants receive adequate training in how to enter these data may ameliorate resistance to tracking and improve overall tracking adherence. Addressing these issues in future research may prove key to improving overall feasibility outcomes.

Despite the issues identified regarding the protocol's feasibility, this study demonstrates the preliminary efficacy of the Mymee intervention. Substantial decreases in pain-related disability, negative emotions and anxiety levels, along with measurable increases in quality of life, positive emotion, and pain self-efficacy scores were observed among intervention participants relative to controls. These findings support the utility of using symptom tracking and analysis along with tailored behavior changes recommended by trained health coaches. There are several lines of evidence that the approach employed in the current study provides a uniquely powerful and low-risk alternative to pharmacologic methods of managing CNCP. It is now generally accepted that changes in gene expression mediated by epigenetic alterations play a role in many illnesses, including cancer (39), autoimmunity (40), and cardiovascular disease (41). Recent investigations suggest that epigenetic changes may also be associated with the development and propagation of CNCP, including symptoms of allodynia, hyperalgesia, anxiety, and depression (42). With the growing understanding that lifestyle factors such as diet (43), sleep habits (44), stress (45), and physical activity (46) affect gene expression via epigenetic mechanisms, it is reasonable to postulate that changes to these modifiable behaviors may lead to beneficial epigenetic alterations and amelioration of CNCP. Several investigators have examined relieving CNCP with alterations in diet (47–49). The ability of specific exercise programs to improve CNCP was the subject of a 2017 Cochrane review, which concluded that, while further research needs to be done, “physical activity and exercise is an intervention with few adverse events that may improve pain severity and physical function, and consequent quality of life” (50). Finally, moderate evidence exists that supports a role for mindfulness-based stress management techniques in the treatment of CNCP (51).

However, understanding which of the myriad lifestyle and eating behaviors need to change on an individual level, and in what fashion, to make meaningful impacts on an individual's health remains challenging. The sheer number of potential environmental determinants of health is daunting and likely varies greatly in each individual. It is the goal of precision medicine to decode this variability in human response to environmental stimuli—behavioral or pharmacological—to treat disease and optimize health most effectively. Digital data capture and analytics provide a potentially powerful way to process the large amounts of information inherent to this precision medicine approach. The Mymee program tested in this pilot study allows users to easily track a multitude of dietary and behavioral variables, as well as daily variation in pain and other symptoms (without the notoriously cumbersome and unreliable method of paper journaling), and enables analysis of the entered data to identify associations between lifestyle and state of health.

Prior studies have examined apps that include tracking and educational components along with health coaching and demonstrated positive results (52–54). Further, a company called Hinge Health has documented impressive improvements in musculoskeletal pain conditions with their platform, which combines digitized, personalized physical therapy with remote patient education and 1-on-1 virtual health coaching (55–57). Our study is the first to employ state-of-the-art data analytics to detect associations between symptoms and lifestyle factors which allows health coaches to make personalized recommendations for behavior change. Collectively, these results support the value of future research designed to determine the value of digital health applications that include a health coaching element.

This study has implications for future research in the area of digital health applications. Studies are needed to identify strategies that can maximize retention and adherence to study procedures (e.g., examining the role of text messaging). A focus on perceived value/benefits of digital applications, prospective participants' motivation for participating in research and technological abilities are all likely factors that impact both engagement and retention in the research process and should be the focus of future studies that enroll older adults (58). Future studies should also examine the degree to which therapeutic alliance (e.g., relationship between the client and health coach) impacts adherence in studies of digital health tools. In addition, research efforts should also explore whether feasibility and efficacy outcomes can be enhanced by involving participants' healthcare providers during or after completion of the intervention.

The current study has several limitations that warrant consideration. First, the convenience sample was small and composed mostly of older women, thereby limiting the external generalizability of our findings. Further, given the difficulties experienced retaining study participants, future work is needed to elucidate how to promote retention of older adults in mHealth studies, particularly studies that require reporting symptom data on a daily basis. Also, we were unable to discern whether the positive effects of the program occurred specifically as a result of diet and lifestyle recommendations made by the health coaches or from the general support and encouragement provided by these coaches.

In conclusion, our study has demonstrated the preliminary efficacy of the Mymee intervention in a sample of community-dwelling older adults with CNCP, while also highlighting the need for more research on ways to optimize retention and adherence outcomes in studies of aging adults with chronic pain. Future research is needed to test approaches (e.g., more education in the early coaching sessions, active involvement of participants' primary care providers) that could positively impact adherence and retention, as well as studies that ultimately confirm the efficacy of the Mymee intervention in larger-scale studies.

## Data availability statement

The raw data supporting the conclusions of this article will be made available by the authors, without undue reservation.

## Ethics statement

The studies involving human participants were reviewed and approved by Weill Cornell Medicine Institutional Review Board (IRB). The patients/participants provided their written informed consent to participate in this study. Written informed consent was obtained from the individual(s) for the publication of any potentially identifiable images or data included in this article.

## Author contributions

MCR and MD conceived and planned the study. UK, CS, and PK assisted with recruitment, data collection, and management of study data. CH performed all analyses. UK, CS, VR, ML, NB, and CH contributed to the interpretation of the results. UK and MR took the lead in co-writing the manuscript. All authors provided critical feedback and helped shape the research, analysis, and manuscript.

## Funding

MCD was supported by grants (P30AG022845, K24AG053462) was from the National Institute on Aging.

## Conflict of interest

Authors MD, VR, ML, and NB were employed by Mymee Inc. The remaining authors declare that the research was conducted in the absence of any commercial or financial relationships that could be construed as a potential conflict of interest.

## Publisher's note

All claims expressed in this article are solely those of the authors and do not necessarily represent those of their affiliated organizations, or those of the publisher, the editors and the reviewers. Any product that may be evaluated in this article, or claim that may be made by its manufacturer, is not guaranteed or endorsed by the publisher.
